# Healthy Subcutaneous and Omental Adipose Tissue Is Associated with High Expression of Extracellular Matrix Components

**DOI:** 10.3390/ijms23010520

**Published:** 2022-01-04

**Authors:** Matúš Soták, Meenu Rohini Rajan, Madison Clark, Christina Biörserud, Ville Wallenius, Carolina E. Hagberg, Emma Börgeson

**Affiliations:** 1 Institute of Medicine, Department of Molecular and Clinical Medicine, Wallenberg Laboratory, Sahlgrenska Academy, University of Gothenburg, 405 30 Gothenburg, Sweden; matus.sotak@wlab.gu.se (M.S.); meenu.rajan@wlab.gu.se (M.R.R.); madison.borgesonlab@gmail.com (M.C.); 2 Wallenberg Centre for Molecular and Translational Medicine, Sahlgrenska Academy, University of Gothenburg, 405 30 Gothenburg, Sweden; 3 Region Vaestra Goetaland, Department of Clinical Physiology, Sahlgrenska University Hospital, 413 45 Gothenburg, Sweden; 4 Region Vaestra Goetaland, Department of Surgery, Sahlgrenska University Hospital, 413 45 Gothenburg, Sweden; christina.biorserud@vgregion.se (C.B.); ville.wallenius@gastro.gu.se (V.W.); 5 Department of Surgery, Institute of Clinical Sciences, Sahlgrenska Academy, University of Gothenburg, 405 30 Gothenburg, Sweden; 6 Division of Cardiovascular Medicine, Department of Medicine Solna, Karolinska Institutet, 171 64 Stockholm, Sweden; Carolina.Hagberg@ki.se; 7 Center for Molecular Medicine, Karolinska Institutet, 171 74 Stockholm, Sweden

**Keywords:** adipose tissue, fibrosis, adipose tissue fibrosis, extracellular matrix, ECM remodeling, obesity, metabolic health, metabolically unhealthy obese, cardiometabolic disease

## Abstract

Obesity is associated with extensive expansion and remodeling of the adipose tissue architecture, including its microenvironment and extracellular matrix (ECM). Although obesity has been reported to induce adipose tissue fibrosis, the composition of the ECM under healthy physiological conditions has remained underexplored and debated. Here, we used a combination of three established techniques (picrosirius red staining, a colorimetric hydroxyproline assay, and sensitive gene expression measurements) to evaluate the status of the ECM in metabolically healthy lean (MHL) and metabolically unhealthy obese (MUO) subjects. We investigated ECM deposition in the two major human adipose tissues, namely the omental and subcutaneous depots. Biopsies were obtained from the same anatomic region of respective individuals. We found robust ECM deposition in MHL subjects, which correlated with high expression of collagens and enzymes involved in ECM remodeling. In contrast, MUO individuals showed lower expression of ECM components but elevated levels of ECM cross-linking and adhesion proteins, e.g., lysyl oxidase and thrombospondin. Our data suggests that subcutaneous fat is more prone to express proteins involved in ECM remodeling than omental adipose tissues. We conclude that a more dynamic ability to deposit and remodel ECM may be a key signature of healthy adipose tissue, and that subcutaneous fat may adapt more readily to changing metabolic conditions than omental fat.

## 1. Introduction

White adipose tissue (WAT) is a highly regulated endocrine organ that plays a critical role in maintaining whole body energy homeostasis. WAT can be compartmentalized into two major anatomically distinct depots (subcutaneous and visceral WAT), further subdivided based on specific locations. Omental fat (oWAT) is the predominant visceral fat depot in humans, whereas the subcutaneous fat (scWAT) of the abdomen is the best studied. These two fat depots are often compared and have been shown to differ in their cellular composition and morphology, secretory profile, and metabolic characteristics [1]. Whereas scWAT is the primary site for lipid storage in healthy humans, oWAT is thought to mainly serve as an immunomodulatory cushion for the gastro-intestinal tract. These depot-specific functions are also reflected by differences in the tissue microenvironment, propensity to inflame, and metabolic regulation [2]. Obesity-induced accumulation of lipids in oWAT is associated with inflammation and a greatly increased risk of developing cardio-metabolic diseases. In contrast, subcutaneous obesity may present a metabolic advantage and even reduce the risk for disease development [3,4,5]. Depending on their fat distribution and adipose tissue functionality, not all obese individuals develop comorbidities such as insulin resistance or hypertension. Likewise, not all lean individuals are free from metabolic disturbances, and may even display the metabolic characteristics associated with obesity. It has become apparent that more knowledge is needed about the pathologies associated with obesity and excessive fat accumulation. A better understanding of the morphological changes associated with diseases within each WAT depot would also allow a better categorization of patients based on metabolic state in the clinic, and enable more targeted development of future therapies within patient subgroups.

WAT is an organ with high plasticity, which can rapidly respond to alterations in energy status by undergoing extensive remodeling and expansion. The adipocytes are surrounded by an extracellular matrix (ECM) that forms a dense three-dimensional network of fibrous proteins e.g., collagens, adhesion proteins, proteoglycans, and modulatory enzymes such as matrix metalloproteinases (MMPs). These proteins provide structural integrity to the tissue and modulate several biological processes, such as cell adhesion, migration, proliferation and differentiation, and mediate signal transduction by sequestering and releasing various growth factors [6]. During weight-gain induced adipose tissue expansion, the ECM needs to be remodeled and reorganized to allow adipocytes to expand within the tissue. ECM accumulation depends on a balance between collagen synthesis and assembly (through the expression of components such as collagens and procollagen C-endopeptidase enhancer (PCOLCE)), and degradation of existing ECM (regulated by MMPs and their inhibitors, called tissue inhibitor of matrix metalloproteinases (TIMPs)) [7]. Healthy expansion of adipose tissue also requires adequate vascularization, regulated mainly by vascular endothelial growth factor A (VEGF-A), which in turn is induced by hypoxia [8]. Thus, an effective adipogenic and angiogenic response with appropriate ECM remodeling is required to maintain metabolically healthy adipose tissue during weight gain [9,10]. However, the question remains what characterizes the ECM of metabolically healthy lean (MHL) individuals compared with those of metabolically unhealthy obese (MUO) patients, and what differences can be found between human oWAT and scWAT fat depots.

Excessive and/or dysregulated WAT expansion triggers many pathological effects such as hypoxia, altered chemokine/adipokine secretion, and ECM deposition. Together, these changes lead to the development of chronic low-grade inflammation in WAT, which ultimately results in systemic metabolic disturbances. One of the long-term consequences of dysregulated WAT remodeling is the development of pathological ECM deposition, often referred to as fibrosis, which is believed to stem from the chronic unresolved inflammation that can develop due to unhealthy WAT expansion [11]. Fibrosis is characterized by excessive accumulation of certain ECM components and modulatory enzymes. The majority of the reports demonstrate high WAT fibrosis in human obesity [12,13,14,15,16], with oWAT being more prone to develop fibrosis than scWAT [12,17]. However, the subject remains controversial as several contradictory reports have been published [18,19,20]. A direct comparison of these studies may be difficult due to differences in the criteria and methodologies used for the assessment of the ECM status and fibrosis level in human fat biopsies. Moreover, the use of biopsies from different anatomical sites and mixed populations of metabolically healthy and unhealthy obese subjects may add additional complexity when comparing reports.

To investigate the detailed quantity and composition of the adipose tissue ECM during health and disease, we studied paired oWAT and scWAT biopsies taken from the same anatomical locations of two distinctly different groups of subjects: MHL and MUO individuals. Using three complementary methods, we show that, at least for the subjects included in this study, MHL typically display more ECM deposition than MUO subjects. MHL also showed higher expression of transcripts associated with ECM remodeling, with the notable exception of lysyl-oxidase (LOX) and thrombospondin 1 (THBS1), which were higher in MUO. In addition, we found many ECM related transcripts to be expressed at higher levels in scWAT as compared with oWAT. Together, our results suggest that healthy adipose tissue is characterized by a higher flexibility that allows for rapid remodeling of the ECM, while the ECM surrounding adipose tissue in obese subjects may be stiffer and less adaptable to changing conditions. 

## 2. Results

### 2.1. Patient Characteristics

We recruited two distinctly different patient groups of MHL and MUO individuals to quantify ECM deposition in human WAT biopsies. A metabolically unhealthy phenotype was defined as displaying three or more of the metabolic syndrome criteria described by the International Diabetes Federation [21] (see methods section for further details). Paired oWAT and scWAT samples were obtained from all participants. Furthermore, the adipose tissue from lean and obese subjects was obtained from the same anatomical subregion. Both patient groups underwent a detailed health exam and were characterized in the clinic by anthropometrical, clinical and biochemical measurements (Table 1). The groups did not differ significantly in age, and both groups included female and male participants, although the patient group size was not sufficiently large to determine the impact of sex. Compared with MHL, body weight, BMI, and waist circumference were higher in MUO, as expected.

Cardiovascular parameters, namely systolic and diastolic blood pressure, as well as serum triglycerides, were significantly higher in MUO, whereas total cholesterol, HDL-cholesterol, and LDL-cholesterol did not differ between the groups. In addition, fasting plasma glucose and HbA1c were also significantly elevated in MUO. A higher level of serum C-reactive protein (CRP) was detected in the MUO group, possibly indicative of systemic low-grade inflammation. From other blood parameters, such as liver enzymes or hematological parameters, alanine transaminase (ALT) levels were significantly higher in MUO compared with MHL. Some of the subjects in the MUO group were taking anti-hypertensive (7/15), diabetes (2/15) or lipid lowering (2/15) medications, whereas no subjects in the MHL group were treated with these medications. Lastly, adipocyte size was determined in both oWAT and scWAT as median adipocyte area for each subject. As expected, adipocyte size within both fat depots was significantly larger for MUO subjects than for MHL subjects (Table 1).

### 2.2. Histological Staining of Collagen in oWAT and scWAT of MHL and MUO Subjects

**Figure 1:**
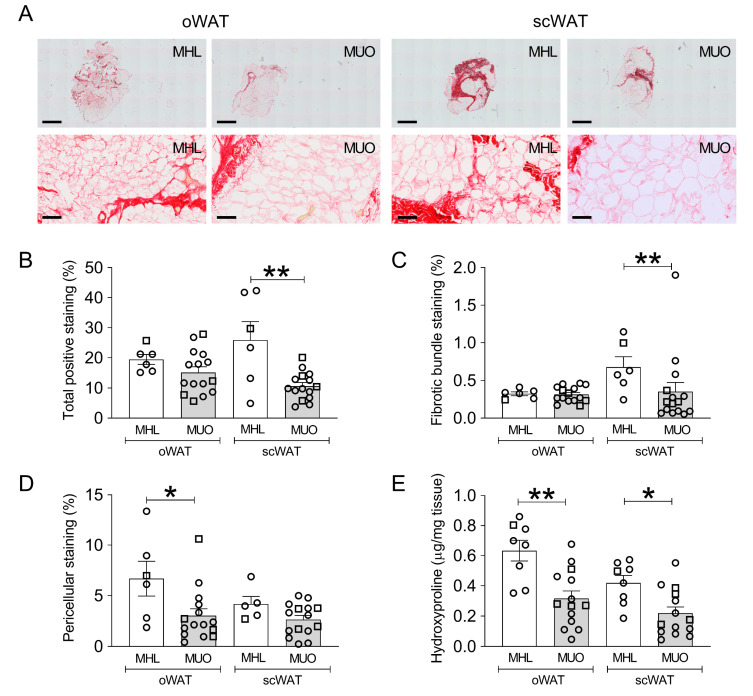
Extracellular matrix (ECM) deposition in omental and subcutaneous white adipose tissue (WAT) of metabolically healthy lean (MHL) and metabolically unhealthy obese (MUO) subjects. (**A**) Representative images for picrosirius red staining. Scale bar for upper row = 2 mm and for the second row = 100 µm. (**B**–**D**) Comparison of total ECM staining intensity (**B**), fibrotic bundle staining (**C**), and pericellular staining (**D**) in omental WAT (oWAT) and subcutaneous WAT (scWAT) of 6 MHL and 15 MUO subjects. Data show the amount of stained area as a percentage of the total area and expressed as mean ± SEM and individual values (○ open circles, females; □ open squares, males); (**E**) Hydroxyproline content in oWAT and scWAT was measured as an indicator of total ECM collagen content in 8 MHL and 14 MUO subjects. Data expressed as mean ± SEM and individual values (○ open circles, females; □ open squares, males). Statistical analysis was performed using Kruskal-Wallis test followed by Dunn’s multiple comparison post-hoc test. * *p* ≤ 0.05, ** *p* ≤ 0.01.

To evaluate total ECM deposition, we performed picrosirius red staining of collagen fibrils on oWAT and scWAT sections from MHL and MUO individuals (Figure 1A). Total ECM content, quantified as the total signal intensity from picrosirius red staining divided by the total tissue area, was not different between the oWAT and scWAT depots either when performing paired analysis of all subjects together (mean ± SEM; oWAT 16.42 ± 1.43, scWAT 15.01 ± 2.41; *p* = 0.320) or when comparing oWAT with scWAT for either of the groups separately (Figure 1B). However, when comparing the patient groups with each other, we found total ECM deposition in scWAT to be significantly higher for MHL subjects than MUO individuals (Figure 1B). The difference in oWAT was not significant. The same pattern could be observed for fibrotic bundle staining, as significantly higher staining was detected for MHL when compared with MUO subjects within scWAT, but not oWAT (Figure 1C). In contrast, we observed significantly higher staining of pericellular fibrosis only in oWAT from MHL individuals compared with MUO individuals (Figure 1D). Again, no difference in pericellular fibrosis was identified between the oWAT and scWAT depots for either patient group, or when performing paired comparisons between depots for all subjects together (mean ± SEM; oWAT 4.18 ± 0.79, scWAT 3.03 ± 0.38; *p* = 0.245).

### 2.3. Total Collagen Content Assessed by Quantification of Hydroxyproline Content

To confirm the findings obtained from quantifying picrosirius red staining on histological sections, we carried out a colorimetric assay to determine the total tissue hydroxyproline content in oWAT and scWAT biopsies. Hydroxyproline, a derivate of the amino acid proline, is a major component of the collagen triple helix, and its presence is largely restricted to collagen deposited within the ECM. It is therefore often used as an indicator of total collagen amount. Measuring hydroxyproline showed higher collagen ECM content in the MHL group than the MUO group for both adipose tissue depots, oWAT and scWAT (Figure 1E). In contrast to the picrosirius red staining, paired analysis comparing the hydroxyproline content in oWAT with that of scWAT for all subjects together did result in a significant difference between depots, with higher levels in oWAT (mean ± SEM; oWAT 0.43 ± 0.05, scWAT 0.29 ± 0.04; *p* < 0.001). When comparing either of the patient groups alone, the difference between depots was not significant (Figure 1E). Taken together, the colorimetric hydroxyproline assay confirmed our results from histological sections, showing that the major difference in adipose tissue ECM content was between patient groups, with MHL individuals showing higher levels of tissue collagen than the MUO group. Furthermore, more ECM was detected in oWAT than scWAT when analyzing all subjects together.

### 2.4. mRNA Expression of Extracellular Matrix Genes

**Figure 2:**
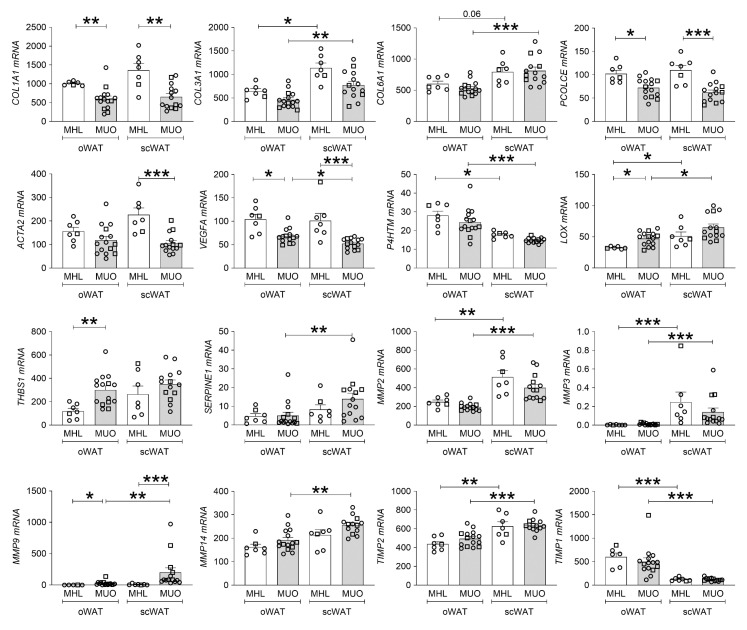
WAT ECM-remodeling gene expression in metabolically healthy lean (MHL) compared with metabolically unhealthy obese (MUO) subjects. mRNA expression data were obtained by ddPCR and calculated as normalized copy number. Data expressed as mean ± SEM and individual values (○ open circles, females; □ open squares, males). Statistical analysis was performed using Kruskal-Wallis test followed by Dunn’s multiple comparison post-hoc test. * *p* ≤ 0.05, ** *p* ≤ 0.01, *** *p* ≤ 0.001.

To further validate our results and quantify the mRNA expression of genes involved in adipose tissue ECM generation and remodeling in MHL and MUO subjects, we performed a droplet digital polymerase chain reaction (ddPCR) (Figure 2). In line with the results from the collagen staining and the hydroxyproline assays showing more ECM in WAT from lean subjects, we found that MHL subjects had a higher transcriptional expression of collagen *COL1A1* and the procollagen cleavage enzyme *PCOLCE* in both fat depots. In contrast, levels of *MMP9*, involved in ECM degradation, were lower compared with MUO subjects. In addition, MHL subjects displayed higher scWAT expression of smooth muscle actin (encoded by the gene *ACTA2*), a marker for myofibroblasts, when compared with MUO. Although no significant difference in expression of the hypoxic sensor prolyl 4-hydroxylase (*P4HTM)* was observed between subject groups, MUO subjects had reduced transcription of the pro-angiogenic factor *VEGFA* in both depots. 

MUO subjects did however express higher mRNA levels of the pro-fibrotic genes lysyl oxidase (*LOX)* and thrombospondin 1 (*THBS1)* in oWAT. LOX is involved in collagen cross-linking and tissue stiffness, while THBS1 is an adhesive glycoprotein that mediates cell-cell and cell-matrix interactions and has been shown to inhibit angiogenesis [22]. These changes are in line with the classical view that obesity leads to fibrosis, especially in the oWAT depot. 

The differences between the oWAT and scWAT depots were also assessed. For both subject groups we found higher subcutaneous than omental expression of collagen *COL3A1* and matrix metallopeptidase *MMP2*, the pro-fibrotic metallopeptidase inhibitor *TIMP2* and the pro-fibrotic collagen cross-linker *LOX*. The expression of *COL6A1*, *MMP9*, *MMP14* and *SERPINE1* was only higher in the scWAT from MUO subjects but not in the lean subjects. The expression of metallopeptidase inhibitor *TIMP1* and the hypoxic marker *P4HTM* was lower in scWAT as compared with oWAT for both subject groups. Taken together, the expression analysis confirmed our previous results, suggesting that ECM components were more highly expressed in WAT from MHL subjects than from MUO subjects. Moreover, the expression analysis showed scWAT to be more likely to express components involved in ECM remodeling than oWAT, suggesting that high expression of ECM remodeling enzymes could have beneficial effects on tissue function.

### 2.5. Correlation between Collagen Content, Clinical Parameters and mRNA Expression

To further evaluate the relationship between ECM markers and metabolic phenotype, we performed correlation analysis between clinical parameters and total hydroxyproline content in oWAT and scWAT, respectively. Hydroxyproline content correlated significantly to the total picrosirius red staining intensity (Spearman’s r_s_ = 0.39, *p* = 0.014). Body weight and BMI were negatively correlated with WAT hydroxyproline content in both fat depots, and waist circumference negatively correlated with hydroxyproline content in oWAT. Spearman’s correlation coefficients and respective *p* values are shown in Table 2. In line with this, a significant negative correlation was determined between hydroxyproline content and adipocyte size in oWAT, whereas the correlation did not reach statistical significance (*p* = 0.092) in scWAT. In addition, diastolic blood pressure displayed a negative correlation with ECM content in both oWAT and scWAT depots. Finally, HDL-cholesterol levels in serum were positively correlated with hydroxyproline content in oWAT, but not scWAT.

## 3. Discussion

In order to design better therapies against obesity-induced metabolic diseases it is important to understand the pathological changes related to obesity, specifically in human adipose tissue. Here, we used a combination of three established techniques (picrosirius red staining, a colorimetric hydroxyproline assay and ddPCR measurement of gene expression) to evaluate ECM deposition in the two major human fat depots, oWAT and scWAT, from MHL and MUO subjects. The subject groups were selected to distinguish between the physiological WAT morphology seen in the MHL group and pathological WAT remodeling associated with obesity and metabolic disease in the MUO group.

### 3.1. ECM Specific Differences between Metabolic Groups

Our results indicate that the MHL group is characterized by robust deposition and expression of ECM components compared with the group of MUO individuals. This finding may appear surprising, as many reports show that obesity is associated with higher WAT fibrosis [12,13,23,24], especially in the oWAT depot. Seminal papers published by the Clément group repeatedly demonstrate that fibrosis was significantly higher in obesity in both human oWAT and scWAT [12,13]. However, it is noteworthy that the role of adipose tissue fibrosis in humans has been more debated in recent years [25]. 

We argue that confounding factors may be important when reflecting upon the differences in results. Previous studies did not separate metabolically healthy and unhealthy obese subjects [13], which may be an important variable to consider. We chose to specifically compare healthy, lean individuals to obese subjects that presented with a metabolically unhealthy phenotype.

Elegant studies by Scherer and colleagues have shown that WAT hypoxia is a known contributor to obesity-related WAT fibrosis in mice [26]. We did not observe higher gene expression of the oxygen sensor *P4HTM*, indicating unaltered HIF1α signaling. However, expression levels of the pro-angiogenic factor *VEGFA* was reduced in MUO. This disconnect between oxygen and VEGF-A signaling pathways has been described before, where HIF1α expression in WAT failed to induce the known target gene *VEGFA* [26]. VEGF-A has also been linked to tissue fibrosis [8,27], and thus the lower gene expression of this gene supports our findings of reduced fibrosis in the MUO group. These data suggest a more complex crosstalk between oxygen sensing pathways, such as HIF1α, pro-angiogenic signaling and WAT fibrosis.

Discrepancies in the field might also be due to different studies quantifying, at least in part, different features: some focusing only on pathological fibrosis, whereas others, such as ours, measure total ECM deposition in the tissue. In general, we find that studies measuring the accumulation of specific, pro-fibrotic collagens, including collagen IV, V and VI, seem to more often report a positive correlation between BMI and WAT fibrosis, underlining the pathologic role of their accumulation [13,16,28,29,30]. Several papers, but far from all, which quantify total WAT ECM either by staining or colorimetric assays have found a negative association between total ECM deposition and subject BMI and/or the metabolic health, in line with our results [18,20,31]. There might also exist other qualitative differences in ECM deposition besides collagen type during WAT expansion. Whereas Spencer et al. found significantly higher collagen V deposition in obese scWAT biopsies compared with lean, elastin in the same obese subjects was found to be lower [16]. Other studies report similar findings to our data. Henegar et al. reported less *COL1A1* expression in the obese population and more expression of the pro-fibrotic collagen cross-linker *LOX* [13]. This manuscript also reported higher expression of the more pro-fibrotic collagens *COL4A1*, *COL5A2* and *COL12A1* [13]. 

Similar to our observations, a study by Divoux et al. identified a positive correlation between ECM deposition in oWAT and HDL-cholesterol levels, and a negative association between ECM staining and adipocyte size in the same depot, suggesting that adipocyte hypertrophy is associated with less ECM deposition. The authors also speculate that reduced ECM in obesity potentially increases the capacity for the cells to enlarge [12]. However, this opposes another hypothesis, whereby higher ECM stiffness in obesity (despite reduced expression of ECM components) mechanically stabilizes the adipocytes but prevents the cells from expanding, contributing to developing a metabolically unhealthy phenotype [32]. Our results demonstrate higher LOX activity in obesity, suggesting enhanced ECM cross-linking and higher adipocyte tissue stiffness. Enhanced LOX activity has been shown to increase adipose tissue stiffness in *ob/ob* mice and obese individuals, which was reversed after weight loss surgery [33].

Our results further highlight that robust expression of components involved in ECM remodeling (such as collagens, PCOLCE and ACTA2) could be a sign of WAT health. We speculate that robust expression of ECM remodeling enzymes might enable WAT to dynamically adapt to various changes in triglyceride accumulation and energy levels, whereas elevated expression of LOX and THBS1 contributes to tissue stiffness and accumulation of more fibrotic ECM components.

Summarized, these results highlight the need for a redefinition of WAT fibrosis, starting with differentiating between total ECM accumulation, which sometimes might have a physiological role, and pathological changes to the WAT microenvironment. In contrast to fibrotic staining techniques, higher WAT stiffness, although difficult to measure in experimental laboratories, seems to most often correlate positively with parameters of metabolic disease, and could be the most rigid feature of unhealthy WAT expansion that should be measured more often [18,31,33]. More techniques to experimentally assess WAT ECM stiffness in adipose tissue fibrosis are therefore warranted. Meta-analyses will also aid to identify specific ECM components involved in physiological and pathological ECM remodeling. We speculate that in the future, detailed investigations of WAT ECM could potentially allow for the development of diagnostic methods to identify these changes in early stages of obesity and, from there, develop interventions that prevent the occurrence of inflammatory processes or oxidative damage associated with obesity and related complications such as type 2 diabetes.

### 3.2. Differences between oWAT and scWAT

Another unexpected result was that we did not find any significant difference in picrosirius red staining when comparing paired oWAT versus scWAT tissue biopsies. This result is somewhat reflected in our expression analyses, where we could only identify higher expression of *COL3A1* in scWAT compared to oWAT, and an higher *COL6A1* expression only in scWAT of MUO individuals. However, we consistently found higher scWAT expression of several genes coding for proteins involved in ECM remodeling and cross-linking compared with oWAT, including *MMP2* or *TIMP2*. These changes indicate that scWAT appears to be more dynamically regulated compared to oWAT. In line with this observation, oWAT showed almost no expression of matrix metalloproteinases *MMP3* and *MMP9*, while displaying high levels of the MMP inhibitor *TIMP1*. Looking at specific substrates for MMP3 and MMP9 revealed that both enzymes target several collagen classes, including collagens III and IV [34]. Collagen III forms the structural framework in adipose tissue, while collagen IV is associated with the formation of the basal lamina, providing structural support for the tissue [35]. Low expression of these MMPs, with higher expression of *TIMP1*, which inhibits nearly all matrix metalloproteinases [36], further supports lower adaptability of the oWAT fat depot compared with scWAT.

### 3.3. Summary

**Figure 3:**
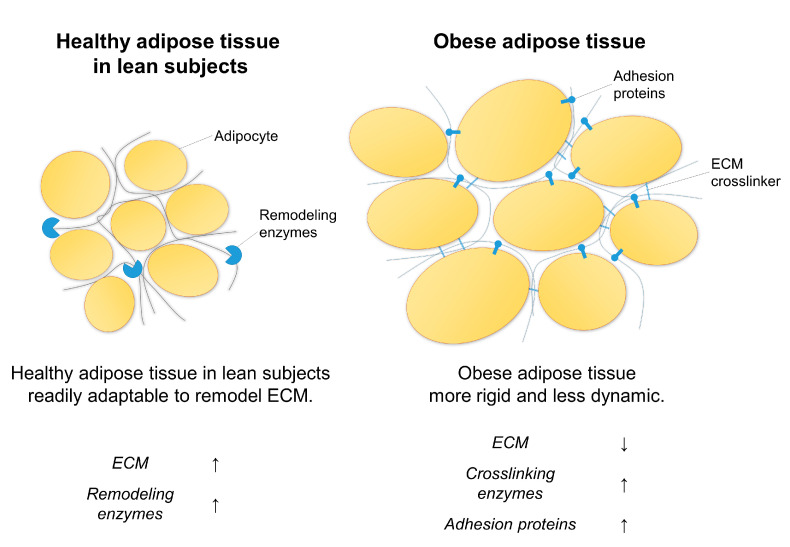
Summarized main findings highlighting the differences between healthy adipose tissue in lean subjects and adipose tissue in metabolically unhealthy obese individuals. ECM, extracellular matrix.

We propose that healthy WAT is characterized by robust ECM deposition and turnover. Conversely, low total ECM content with higher rigidity might be a characteristic of obese, unhealthy WAT in humans (Figure 3). Our results align with the hypothesis that in the lean state, the ECM of WAT acts favorably to allow dynamic expansion of adipocytes, despite higher collagen levels. A reduction in ECM remodeling by higher collagen cross-linkage by LOX and enhanced cell-cell and cell-matrix interactions through THBS1, lead to developing WAT dysfunction that characterizes unhealthy obesity. 

Taken together, our results measuring the ECM status in MHL and MUO subjects underline the marked difference between physiological ECM accumulation and pathological fibrosis. Data from our experiments also highlight the importance of understanding what exactly the measured parameters are quantifying. Part of the discrepancy between the results reported here and the current view of the field might stem from differences in recruited patients, techniques used, and genes measured. However, our report is by no mean the first to find a negative association between WAT collagen content and patient BMI, and highlights the need for further studies and in-depth discussions. Our data also suggest that quantifying WAT stiffness and remodeling might be better measurements of the pathological state associated with obesity than picrosirius red staining alone.

### 3.4. Study Limitations

Several limitations may be taken into consideration when interpreting our study results. First, the number of participants is limited (*n* = 8 and 15 in MHL and MUO groups, respectively), although the groups were carefully characterized and multiple methods applied to analyze the ECM deposition. Although both males and females were included in the study, the sexes were not equally distributed as females were overrepresented in both groups. Hence, our analyses do not allow for a sex-dependent statistical evaluation. This is an important consideration, as reports show that males and females have differences in adipose tissue physiology and pathophysiology [37]. However, when presenting our data as individual data points where the sex of each individual is shown, we could not observe any apparent skewness of the data due to sex. Adipose tissue biology is also affected by hormones such as estrogen [38]. None of the participants included in these analyses received hormonal or contraceptive treatment, however, we did not collect the information about the menopausal status of the participants. 

The extreme differences in fat mass and adipocyte size between lean and obese subjects make it difficult to directly compare extracellular content with suitable normalization strategy. We normalized values according to widely used strategies in the field (e.g., picrosirius red staining normalized to tissue area, ECM content in relation to tissue weight, and RNA expression in relation to housekeeping genes that show stable expression in human adipose tissue) [12,13,20,39,40]. This makes our results comparable with most published studies in the field. However, further studies that use larger patient cohorts and characterize the relative influence of ECM quantification in relation to various normalization strategies (including that of adipocyte cell size) would represent a valuable addition to the field.

Another important consideration is that gastric bypass patients underwent a pre-surgical diet prior to tissue collection and we cannot exclude the possibility that this may have an affect (e.g., in our transcriptome analyses). However, previous studies by Clément and colleagues have shown that short-term caloric restriction increases adipose tissue ECM deposition in obese gastric bypass patients [41]. Thus, the trend shown in our study whereby lean subjects have more ECM as compared with obese subjects would likely be even more pronounced if analyzing adipose tissue obtained prior to the pre-surgical diet.

Finally, another limitation is that since we did not investigate protein expression in this study, we do not know if the statistically significant differences in gene expression with various size effects translate into protein expression and protein function. Although the effect size is rather small in the expression of some genes, it can still translate into significant functional differences, especially in the case of enzymes.

## 4. Materials and Methods

### 4.1. Study Participants

Obese subjects [body mass index (BMI) median and interquartile range (IQR) 43.2 (39.6–44.9) kg/m^2^, aged 47.0 (35.0–54.0) years] were recruited from a group of patients scheduled to undergo laparoscopic gastric bypass surgery and lean controls [BMI, median and IQR 22.2 (21.7–23.2) kg/m^2^, aged 40.0 (33.8–47.2) years] were recruited from patients scheduled for non-acute cholecystectomy due to uncomplicated bile stone disease. The volunteer patients were subsequently characterized as metabolically healthy or unhealthy, based on the International Diabetes Federation (IDF) criteria of the metabolic syndrome [21]. Thus, obese subjects included in the study displayed three or more metabolic syndrome components in order to be characterized as MUO and match the recruitment criteria.

Importantly, fat biopsies collected during gastric bypass and cholecystectomies were taken from the same anatomical subregions, which is pivotal when comparing patients undergoing different types of surgeries. 

MUO subjects scheduled for gastric bypass underwent short-term low-calorie diet restriction three weeks before surgery. Therefore, the anthropometric measurements and blood collection were performed before the start of the pre-surgical dieting, to avoid the effect of short-term calorie restriction. For MHL subjects, all samples were collected on the day of surgery. Subjects were excluded if they were taking anti-inflammatory and/or immunosuppressive drugs, currently smoked, or were diagnosed with significant gastrointestinal disease or inflammatory bowel disease.

The study was approved by the Swedish Ethical Review Authority #682-14 (ClinicalTrials.gov NCT02322073). All procedures were carried out in accordance with the Helsinki Declaration and written informed consent was obtained from all participants.

### 4.2. Plasma and Serum Preparation and Biochemical Laboratory Analyses

Certified nurses collected venous blood into K_2_EDTA tubes (#456243, Greiner Bio-One, Kremsmünster, Austria) for anticoagulated blood or plasma preparation and Z Serum Separator Clot Activator tubes (#454420, Greiner Bio-One) for serum, as previously described [42]. Hematological and biochemical parameters of blood, plasma or serum were assessed by an accredited hospital laboratory (Sahlgrenska University Hospital, Gothenburg, Sweden).

### 4.3. Adipose Tissue Samples

Paired oWAT and scWAT samples were obtained from MHL and MUO subjects from the respective sites during surgery and immediately processed further. Tissue samples for RNA analysis and the hydroxyproline assay were snap frozen in liquid nitrogen and stored at −80 °C before analysis. Moreover, samples were also fixed in 4% formaldehyde and processed for paraffin embedding for immunohistochemistry.

### 4.4. Immunohistochemistry Analysis

Paraffin embedded formaldehyde fixed oWAT and scWAT biopsies were sectioned (5 µm/section) and stained with Picrosirius Red (Histocenter, Gothenburg, Sweden; accredited by Swedac 17,025:2005). Images were acquired at 20× magnification on the Metafer Slide Scanning Platform (Altlussheim, Germany) and image analysis was carried out using Aperio ImageScope software (version. 12.4.0.7018). Total fibrosis was quantified as percentage of picrosirius red stained tissue area divided by the total tissue area, as described by Henegar et al. [13]. Similarly, pericellular fibrosis was determined by examining the picrosirius red staining in 2–10 randomly chosen 10× fields in parts of the tissue free of fibrotic bundles and expressed as the percentage of the sum of stained areas divided by the total sum of field surfaces. The semi-automatic image analysis using a manually optimized algorithm classified positively stained pixels into strong positive, positive and weak positive. Fibrotic bundle staining was determined by taking the ratio of strong positively stained pixels and dividing them by the positively + weakly positive stained pixels.

To estimate adipocyte size, intact adipocytes were manually circled and the median adipocyte area was calculated using the Aperio ImageScope analysis software (version. 12.4.0.7018). 

### 4.5. Hydroxyproline Assay

Hydroxyproline content was measured using a hydroxyproline assay kit (#MAK008, Sigma-Aldrich, St. Louis, MO, USA). Briefly, 20 mg frozen adipose tissue pieces were weighed and homogenized in 200 µL water. 100 µL of the lysate was then incubated with 100 µL 12 M HCl at 120 °C for 3 h. 10 µL of the hydrolyzed samples were dried before incubation with chloramine-T and p-dimethyl-amino-benzaldehyde at 60 °C for 90 min. Absorbance was measured at 560 nm, and the concentrations of the samples were determined using a standard curve generated with hydroxyproline provided in the kit.

### 4.6. RNA Isolation

Frozen adipose tissue (approximately 100 mg) was homogenized in 1 mL of Trizol reagent (#15596018, Invitrogen, Waltham, MA, USA) with 5 mm Stainless Steel Bead (#69989, Qiagen, Hilden, Germany) using TissueLyser (Qiagen). After 10 min incubation, the homogenate was centrifuged (12,000× *g*). According to the manufacturer’s instructions, the top lipid layer was discarded and 0.8 mL of supernatant was used for subsequent chloroform extraction until an upper water phase with RNA was formed and transferred to the new tube. An equal volume of 70% ethanol was added and mixed thoroughly. The mixture was transferred to RNeasy Mini Kit column (#74106, Qiagen), and total RNA was isolated according to the manufacturer’s instructions. RNA was eluted in 30 µL of PCR grade water. Concentration and purity of RNA were assessed using NanoDrop 2000 (ThermoFisher Scientific, Waltham, MA, USA) and RNA integrity was analyzed using RNA Pico Chips (#5067-1513, Agilent Technologies, Waldbronn, Germany) and the Agilent 2100 Bioanalyzer (Agilent Technologies).

### 4.7. Reverse Transcription and Droplet Digital Polymerase Chain Reaction

0.5 µg total RNA was treated with DNase I (#18068015, Invitrogen) and first strand cDNA was synthesized in 20 µL reaction volume using random primers (#48190011, Invitrogen), dNTP mix, DTT, RNase inhibitor (#10777019, Invitrogen), and SuperScript IV Reverse Transcriptase (#18090050, Invitrogen). Gene expression was evaluated by droplet digital PCR (ddPCR) using QX200 AutoDG Droplet Digital PCR System (#1864100, Bio-Rad, Hercules, CA, USA). 22 µL of PCR reaction mix comprised of ddPCR Supermix for Probes without dUTP (#1863023, Bio-Rad), specific target (FAM-labeled) and reference (HEX-labeled) PrimePCR ddPCR Expression Probe Assay (Table S1) and 2 µL of appropriately diluted template cDNA (3×, or 30×, respectively). Single droplets were generated in DG32™ Automated Droplet Generator Cartridges (#1864108, Bio-Rad) using Automated Droplet Generator (#1864101, Bio-Rad) and Automated Droplet Generation Oil for Probes (#1864110, Bio-Rad). PCR reaction was run in C1000 Touch Thermal Cycler (Bio-Rad) according to the manufacturer’s instructions (95 °C for 10 min for enzyme activation, 40 cycles of denaturation at 94 °C for 30 s followed by annealing and extension at 60 °C for 1 min, 98 °C for 10 min for enzyme denaturation, 4 °C overnight). Droplet reading was performed using the QX200 Droplet Reader (#1864003, Bio-Rad), and data was analyzed using QuantaSoft software (ver. 1.7.4, Bio-Rad). The same manual threshold settings were applied for each specific target assay across all samples. The absolute number of mRNA copies per µL was assessed, and the value per 1 ng of input total RNA was calculated for each target and reference gene. For gene expression normalization, two reference internal control genes (*LRP10*, *RPLP0*) stably expressed in human adipose tissue [39] were selected, and the normalization factor was calculated as a geometric mean of their values normalized to maximal RNA copy value of each gene [43]. Normalized copy number was calculated as absolute mRNA copy number per 1 ng RNA divided by the normalization factor. When the group consisted of *n* ≥ 6, Grubb’s test was used on log_2_ values to identify outliers.

### 4.8. Statistical Analyses

Spearman’s correlation tests were used to calculate the Spearman’s rank correlation coefficient (r_s_) and *p* values between clinical parameters and the hydroxyproline content or mRNA expression in oWAT and scWAT, respectively. MHL and MUO groups were pooled to allow a sufficient number for correlation analysis. There were missing values for some clinical parameters, and these were excluded as only paired values were included in the correlation analysis for each specific parameter.

The differences in clinical parameters or adipocyte size between MHL and MUO were assessed using the Mann-Whitney U test. Fibrosis quantification, hydroxyproline content and mRNA expression were analyzed using the Kruskal-Wallis test with Dunn’s post-hoc pairwise test. To assess difference between oWAT and scWAT in total fibrosis, pericellular fibrosis and hydroxyproline content, the Wilcoxon signed-rank test was used for matched samples. *p* values ≤ 0.05 were considered statistically significant. Analyses were performed in GraphPad Prism (ver. 8.4.3) or R environment (R ver. 4.1.0).

## 5. Conclusions

We found robust ECM deposition in MHL subjects with a higher expression of several components implicated in ECM production and remodeling. MUO individuals displayed lower expression and deposition of ECM components but showed higher expression of genes coding cross-linking and adhesion proteins. We conclude that high ECM deposition and remodeling might be a key signature of healthy adipose tissue. Our study also suggests that subcutaneous fat is generally more adaptable than omental adipose tissue, owing to the higher expression of proteins involved in ECM remodeling. Further studies are needed to understand what differentiates physiological ECM deposition from pathological fibrosis, and what methods are most suited to measure the two conditions in human fat.

## Figures and Tables

**Table 1:** Patient characteristics. Data are presented as the median and interquartile range (25–75%). The differences between metabolically healthy lean (MHL) and metabolically unhealthy obese (MUO) groups were assessed using Mann–Whitney U test for numerical values, and Fisher’s exact test for categorical variables. * *p* ≤ 0.05; ** *p* ≤ 0.01; *** *p* ≤ 0.001.

Variables	MHL	MUO	*p* Values	Significance
Characteristics				
*n*	8	15		
Sex	1 ♂/7 ♀	4 ♂/11 ♀		
Age (years)	40.0 (33.8–47.2)	47.0 (35.0–54.0)	0.583	
Anthropometrics				
Body weight (kg)	64.0 (59.2–65.5)	131.5 (113.8–138.0)	0.0001	***
BMI (kg/m^2^)	22.2 (21.7–23.2)	43.2 (39.6–44.9)	<0.0001	***
Waist circumference (cm)	80.0 (77.5–88.0)	124.5 (120.0–129.5)	0.0001	***
Clinical measurements				
BP diastolic (mm Hg)	72.5 (69.5–77.0)	88.0 (81.0–96.0)	0.002	**
BP systolic (mm Hg)	119.0 (113.5–123.5)	139.0 (132.5–148.5)	0.0009	***
Heart Rate (bpm)	79.5 (76.5–81.8)	83.0 (69.5–89.5)	0.815	
Triglycerides (mmol/L)	0.8 (0.7–0.9)	1.2 (1.0–1.6)	0.024	*
Cholesterol (mmol/L)	4.6 (4.5–5.2)	4.8 (4.2–5.0)	0.569	
HDL-cholesterol (mmol/L)	1.4 (1.3–1.6)	1.2 (1.0–1.2)	0.122	
LDL-cholesterol (mmol/L)	3.3 (3.1–3.6)	3.0 (2.8–3.2)	0.227	
Glucose (mmol/L)	5.6 (5.1–5.9)	6.5 (5.9–7.0)	0.012	*
HbA1c (mmol/mol)	30.0 (27.5–31.5)	37.0 (34.0–42.0)	0.024	*
ALT (µkat/L)	0.3 (0.3–0.4)	0.5 (0.4–0.7)	0.033	*
ALP (µkat/L)	1.1 (1.0–1.4)	1.3 (1.0–1.6)	0.456	
AST (µkat/L)	0.3 (0.3–0.4)	0.4 (0.3–0.4)	0.608	
GGT (µkat/L)	0.3 (0.2–0.4)	0.5 (0.4–1.0)	0.100	
CRP (mg/L)	1.0 (1.0–1.0) ^#^	5.5 (4.0–7.5)	0.025	*
Total leukocytes (10^9^/L)	7.4 (5.6–7.7)	6.5 (6.1–8.2)	0.906	
Platelets (10^9^/L)	235.0 (196.0–253.5)	255.0 (229.5–299.0)	0.286	
APTT (sec)	27.0 (25.8–29.5)	29.0 (26.0–31.0)	0.913	
Prothrombin complex (INR)	1.0 (0.9–1.1)	0.9 (0.9–1.0)	0.118	
Hemoglobin (g/L)	140.5 (133.0–143.0)	142.0 (129.0–145.5)	0.796	
Bilirubin (µmol/L)	6.1 (3.9–9.8)	5.3 (4.3–6.4)	0.675	
Creatinine (µmol/L)	63.0 (59.0–65.5)	68.0 (61.0–79.5)	0.441	
Adipocyte size oWAT (µm^2^)	1838 (1658–2004)	5041 (4582–5640)	<0.0001	***
Adipocyte size scWAT (µm^2^)	3327 (1938–3987)	5510 (4776–6063)	0.0006	***
Medications				
Diabetes medications	0% (0/8)	13.3% (2/15)	0.526	
Anti-hypertensive medications	0% (0/8)	46.7% (7/15)	0.052	
Lipid lowering medications	0% (0/8)	13.3% (2/15)	0.526	

^#^ To enable statistical analysis of C-reactive protein (CRP), values which were below the limit of detection (LOD = 1) were replaced with LOD values. ♂, males; ♀, females. Abbreviations: BMI, body mass index; BP, blood pressure; HbA1c, glycated hemoglobin; HDL, high-density lipoprotein; LDL, low-density lipoprotein; ALP, alkaline phosphatase; AST, aspartate transaminase; ALT, alanine transaminase; GGT, gamma-glutamyl transferase; APTT, activated partial thromboplastin time; INR, international normalized ratio; oWAT, omental white adipose tissue; scWAT, subcutaneous white adipose tissue.

**Table 2:** Correlation analysis between clinical parameters and hydroxyproline content (µg/mg tissue) in oWAT and scWAT, respectively. r_s_, Spearman’s rank correlation coefficient; *p* value, the statistical significance of Spearman’s correlation test. Blue and red colors indicate a significant negative and positive correlation, respectively.

	oWAT ECM		scWAT ECM	
	r_s_	*p* Value	r_s_	*p* Value
Age	0.238	0.286	0.055	0.806
Body weight	−0.585	0.004	−0.465	0.029
BMI	−0.621	0.003	−0.461	0.032
Waist circumference	−0.569	0.006	−0.419	0.052
BP systolic	−0.414	0.055	−0.252	0.258
BP diastolic	−0.792	<0.001	−0.504	0.017
Heart Rate	−0.311	0.183	−0.308	0.186
Glucose	−0.176	0.471	−0.271	0.262
HbA1C	−0.128	0.625	0.169	0.518
Cholesterol	0.365	0.150	0.180	0.490
HDL-cholesterol	0.481	0.050	0.163	0.532
LDL-cholesterol	0.333	0.192	0.203	0.435
Triglycerides	−0.269	0.297	−0.025	0.926
CRP	−0.269	0.296	−0.090	0.732
ALP	−0.150	0.506	−0.044	0.845
AST	0.018	0.936	0.164	0.465
ALT	−0.293	0.185	−0.126	0.576
GGT	−0.160	0.540	−0.053	0.840
Total leukocytes	−0.206	0.427	0.026	0.922
Platelets	−0.372	0.142	−0.343	0.177
APTT	−0.061	0.800	−0.211	0.372
Prothrombin complex	0.164	0.489	0.156	0.512
Hemoglobin	−0.058	0.797	−0.226	0.312
Bilirubin	0.011	0.960	−0.291	0.189
Creatinine	−0.023	0.929	−0.177	0.497
Adipocyte size (median)	−0.629	0.004	−0.388	0.092

Abbreviations: BMI, body mass index; BP, blood pressure; HbA1c, glycated hemoglobin; HDL, high-density lipoprotein cholesterol; LDL, low-density lipoprotein cholesterol; ALP, alkaline phosphatase; AST, aspartate transaminase; ALT, alanine transaminase; GGT, gamma-glutamyl transferase; APTT, activated partial thromboplastin time.

## Data Availability

Data are contained within the article or Supplementary Material.

## Supplementary Materials

The following supporting information can be downloaded at: https://www.mdpi.com/article/10.3390/ijms23010520/s1.
