# Supplementary material for: Healthy Subcutaneous and Omental Adipose Tissue Is Associated with High Expression of Extracellular Matrix Components
Source: Int J Mol Sci. 2022 Jan 4;23(1):520. doi: 10.3390/ijms23010520 (PMC8745535; doi:10.3390/ijms23010520)
Supplement: Supplementary file 1 [file ijms-23-00520-s001.zip › ijms-1466754-supplementary-for publish.pdf]

International Journal of *Molecular Sciences*

MDPI

Supplementary Materials

Healthy Subcutaneous and Omental Adipose Tissue Is Associated with High Expression of Extracellular Matrix Components

Matúš Soták [1,2,3,†], Meenu Rohini Rajan [1,2,3,†], Madison Clark [1,2], Christina Biörserud [4,5], Ville Wallenius [4,5], Carolina E Hagberg [6,7] and Emma Börgeson [1,2,3,*]

[1] Institute of Medicine, Department of Molecular and Clinical Medicine, Wallenberg Laboratory, Sahlgrenska Academy, University of Gothenburg, SE-40530 Gothenburg, Sweden; matus.sotak@wlab.gu.se (M.S.); meenu.rajan@wlab.gu.se (M.R.R.); madison.borgesonlab@gmail.com (M.C.)
[2] Wallenberg Centre for Molecular and Translational Medicine, Sahlgrenska Academy, University of Gothenburg, 405 30 Gothenburg, Sweden
[3] Region Vaestra Goetaland, Department of Clinical Physiology, Sahlgrenska University Hospital, 413 45 Gothenburg, Sweden
[4] Region Vaestra Goetaland, Department of Surgery, Sahlgrenska University Hospital, 413 45 Gothenburg, Sweden; christina.biorserud@vgregion.se (C.B.); ville.wallenius@gastro.gu.se (V.W.)
[5] Department of Surgery, Institute of Clinical Sciences, Sahlgrenska Academy, University of Gothenburg, 405 30 Gothenburg, Sweden
[6] Division of Cardiovascular Medicine, Department of Medicine Solna, Karolinska Institutet, 171 64 Stockholm, Sweden; Carolina.Hagberg@ki.se
[7] Center for Molecular Medicine, Karolinska Institutet, 171 74 Stockholm, Sweden
* Correspondence: emma.borgeson@wlab.gu.se; Tel.: +46-31-3423833
† Authors contributed equally.

Table S1. List of ddPCR assays used for gene expression quantification.

Gene	NCBI Gene ID	Assay ID (Bio-Rad)
ACTA2	59	dHsaCPE5051320
COL1A1	1277	dHsaCPE5034390
COL3A1	1281	dHsaCPE5040292
COL6A1	1291	dHsaCPE5037568
LOX	4015	dHsaCPE5034786
MMP2	4313	dHsaCPE5033158
MMP3	4314	dHsaCPE5046568
MMP9	4318	dHsaCPE5050120
MMP14	4323	dHsaCPE5038836
P4HTM	54681	dHsaCPE5041634
PCOLCE	5118	dHsaCPE5049504
SERPINE1	5054	dHsaCPE5034214
THBS1	7057	dHsaCPE5037120
TIMP1	7076	dHsaCPE5033820
TIMP2	7077	dHsaCPE5058146
VEGFA	7422	dHsaCPE5034756
LRP10	26020	dHsaCPE5043185
RPLP0	6175	dHsaCPE5031575
